# Supplementary material for: Comparison of Different Pneumorrhaphy Methods after Partial Pulmonary Lobectomy in Dogs
Source: Animals (Basel). 2023 Aug 28;13(17):2732. doi: 10.3390/ani13172732 (PMC10486489; doi:10.3390/ani13172732)
Supplement: Supplementary file 1 [file animals-13-02732-s001.zip › animals-2520344-supplementary.pdf]

Supplementary Material

Comparison of Different Pneumorrhaphy Methods After Partial Pulmonary Lobectomy in Dogs

Paloma Helena Sanches da Silva*, Carlos Eduardo Bastos Lopes, Larissa Bueno Stallmach, Lucas de Oliveira Ferreira, Pedro Antônio Bronhara Pimentel, Antonio Giuliano, Patrícia Maria Coletto Freitas, Rodrigo dos Santos Horta

*** Correspondence:**
Corresponding Author
agiulian@cityu.edu.hk; rodrigohorta@ufmg.br

1 Supplementary Table

Table S1. Values referring to extravasation pressures in mmHg by type of lobar synthesis (mmHg).

	Cobbler suture + simple continuous suture<br>G1	Overlapping continuous suture + simple continuous suture<br>G2	Ford interlocking suture<br>G3	Staples<br>G4	Synthetic glue<br>G5
Lung lobe 1	20	18	15	10	60
Lung lobe 2	20	20	24	22	40
Lung lobe 3	18	20	14	19	20
Lung lobe 4	30	23	19	14	40
Lung lobe 5	14	16	14	18	40
Lung lobe 6	20	20	21	20	80

2 Supplementary Figures

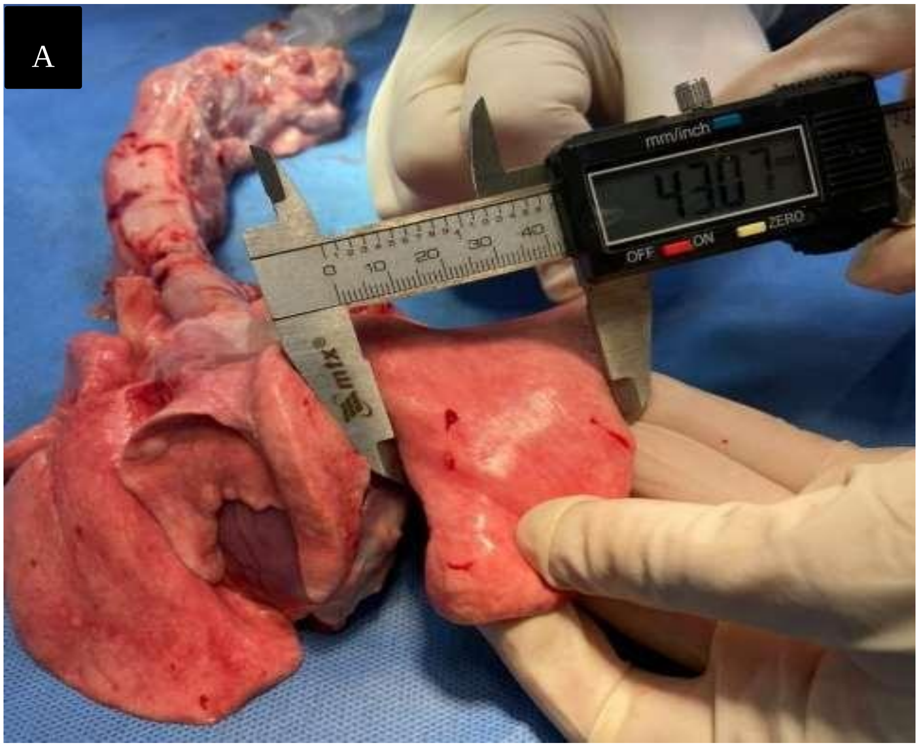

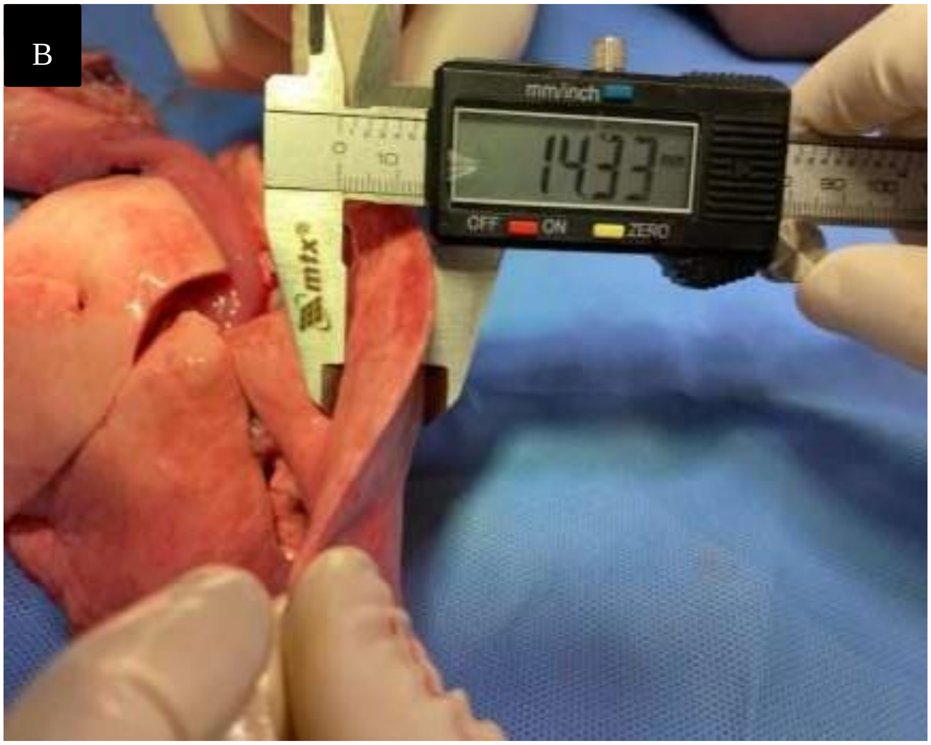

Supplementary Figure S1. A. Measurement of the width of the middle third of the right caudal pulmonary lobe of a dog, using a digital caliper; **B.** Measurement of the height (thickness) of the middle third of the dog's right caudal pulmonary lobe, using a digital caliper.

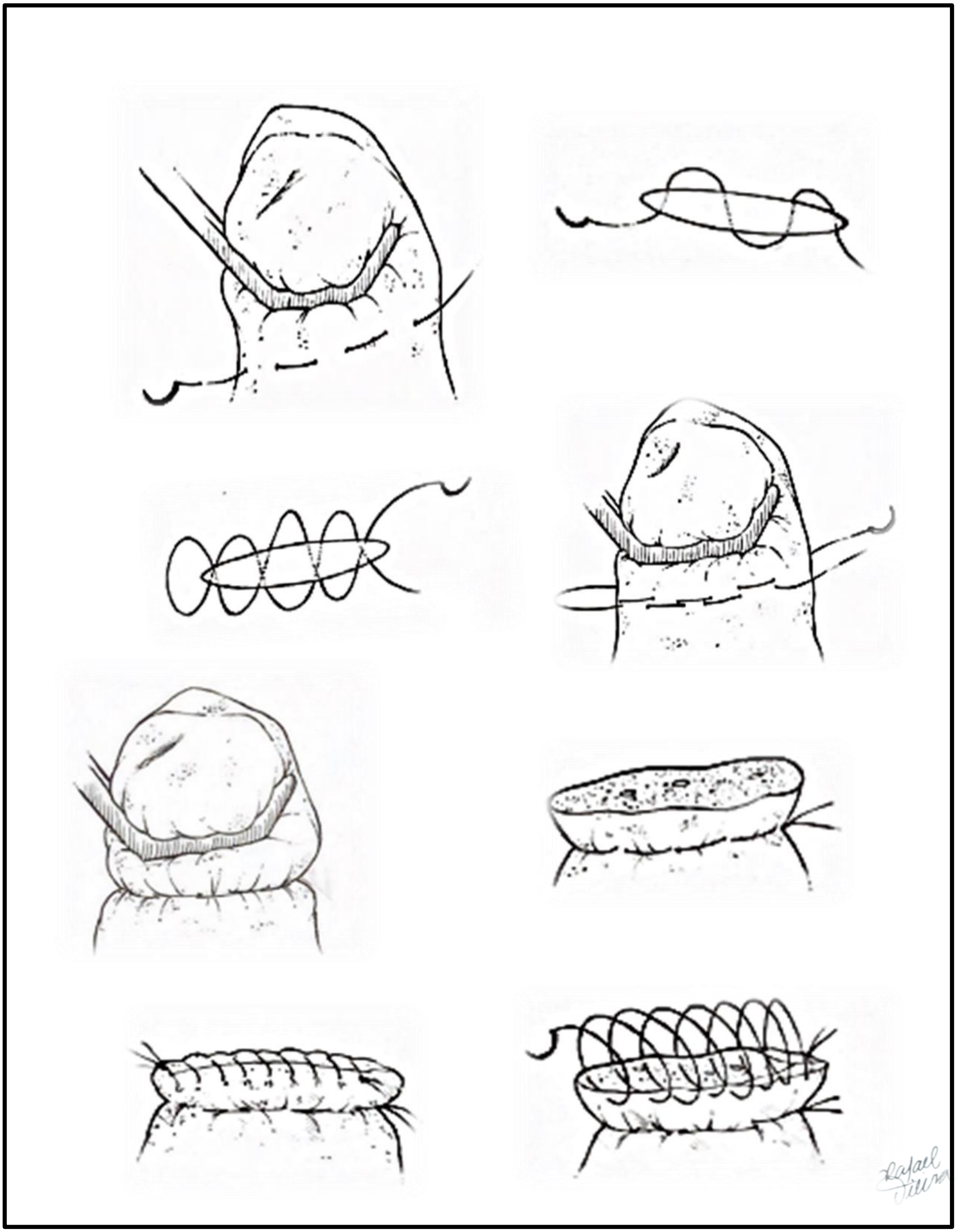

Supplementary Figure S2 - Illustrative drawings demonstrating the steps for making the cobbler manual suture in a dog's lung lobe.

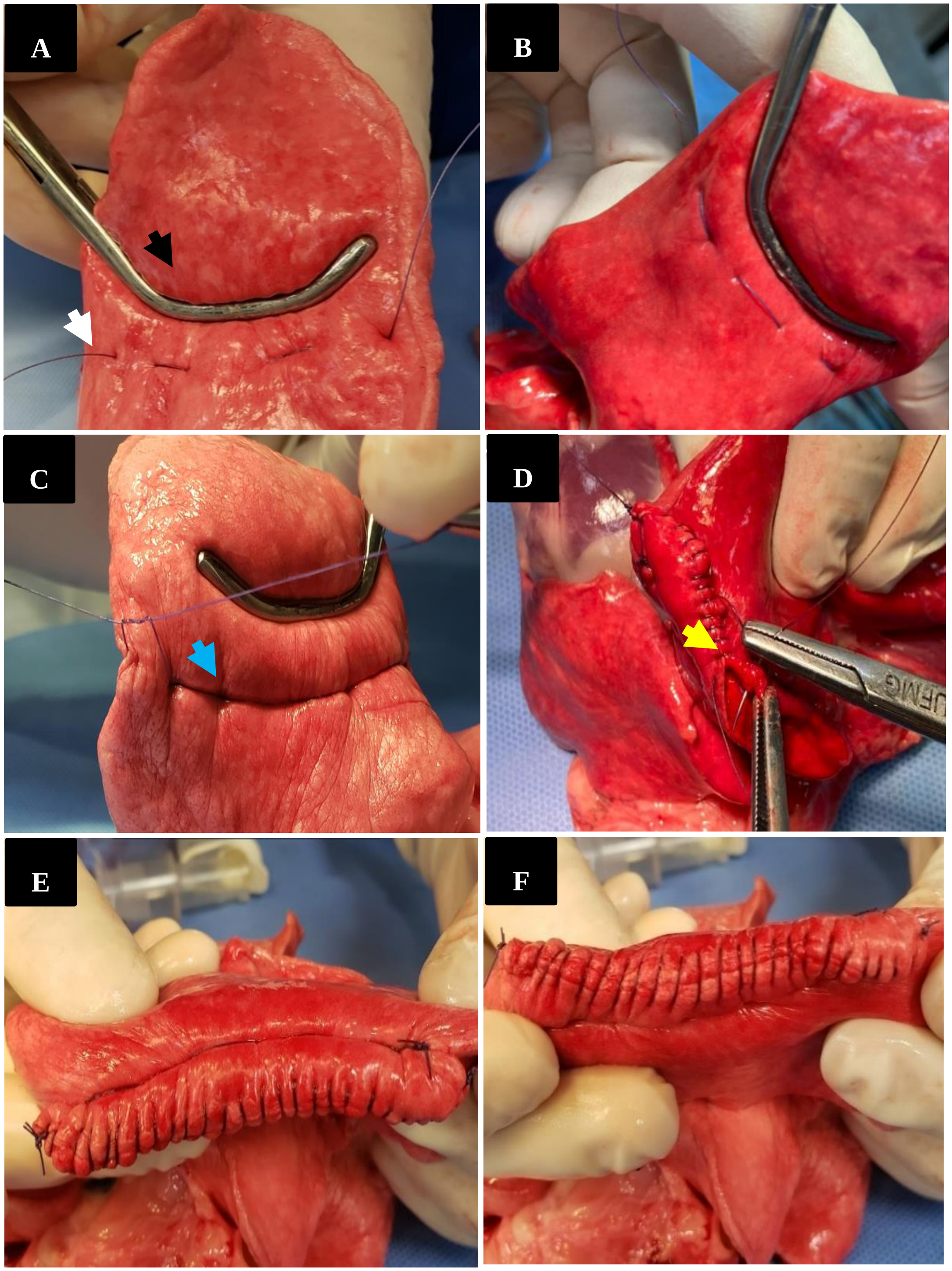

Supplementary Figure S3 - Photographic image of pulmonary suture using cobbler suture associated with simple continuous suture in an anatomical piece of dog lung; A and B. Note the clamping in the middle third of the right caudal lung lobe with a Satinsky forceps (black arrow) and the beginning of the shoemaker suture with Polyglactin 910 thread (white arrow); C. Note the cobbler suture (blue arrow) performed in the lung parenchyma; D. Performing the simple continuous suture (yellow arrow) approaching the visceral pleura, after finishing the shoemaker suture; E and F. The final appearance of the cobbler suture can be observed withtissue

compression and edges of the remaining parenchyma coapted by the simple continuous suture.

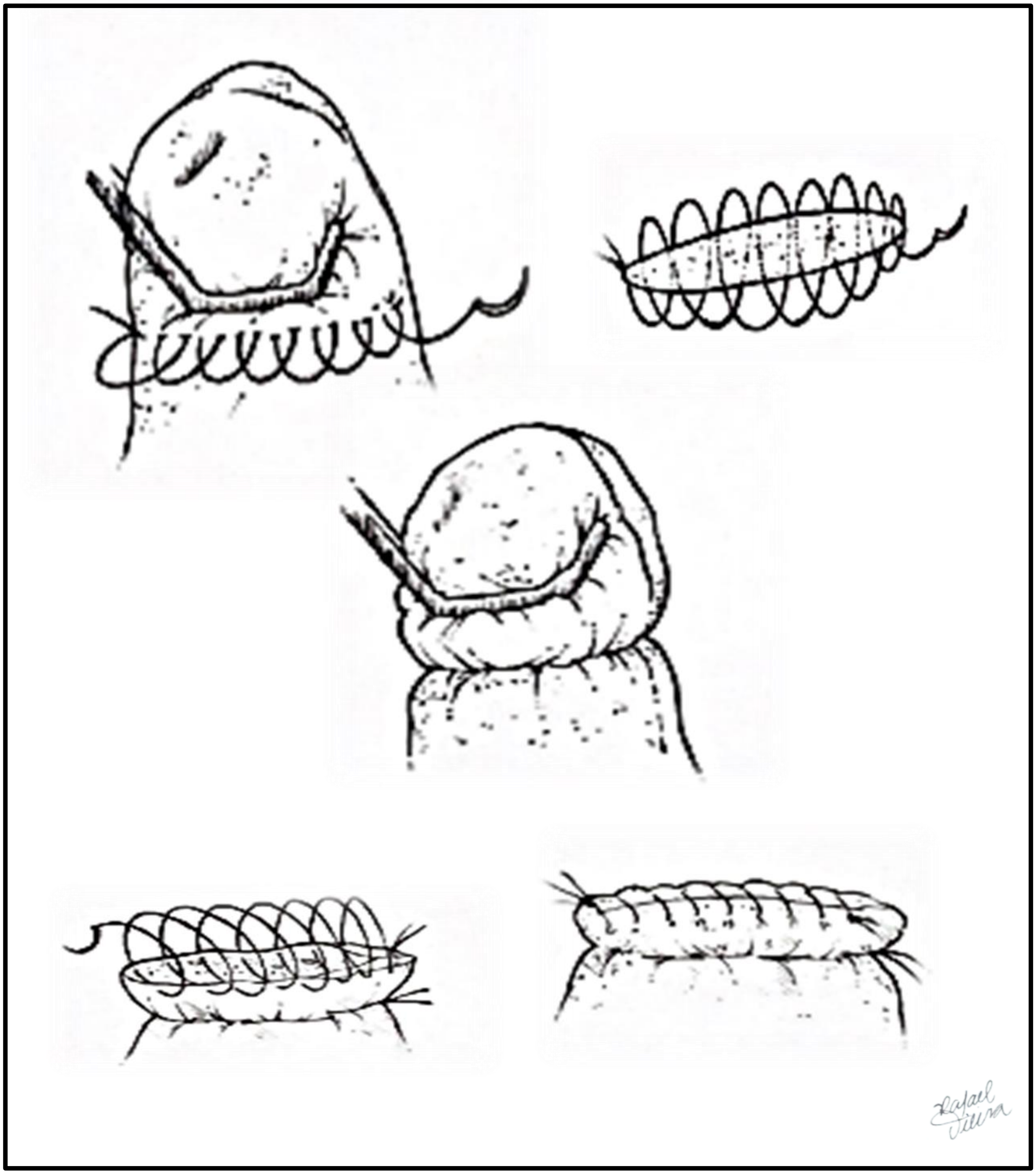

Supplementary Figure S4 - Illustrative drawings demonstrating the steps for making the overlapping continuous suture associated with simple continuous suture on the lung lobe of a dog.

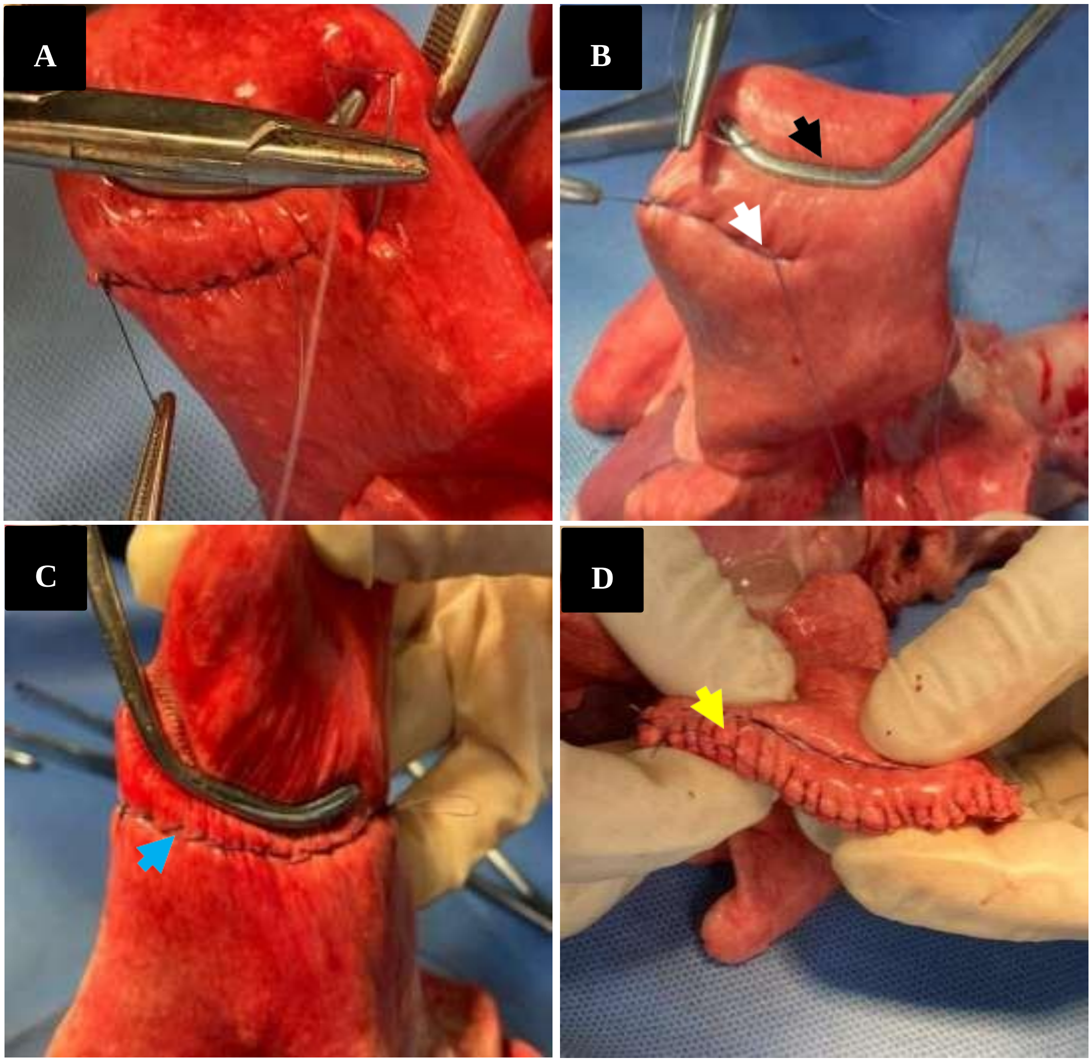

Supplementary Figure S5 - Photographic image of pulmonary suture using overlapping continuous suture associated with simple continuous suture in an anatomical piece of a dog lung; A and B. Note the clamping inthe middle third of the right caudal lung lobe with a Satinsky clamp (black arrow) and making overlapping continuous suture with Polyglactin 910 thread (white arrow) proximally to the clamp; C. Note the overlapping of the thread in the tissue (blue arrow) up to the contralateral end of the lobe after finishing the superimposed continuous suture performed in the parenchyma; D. Final aspect of the coaptation promoted by the apposition of the edges of the remaining parenchyma in a simple continuous suture pattern (yellow arrow).

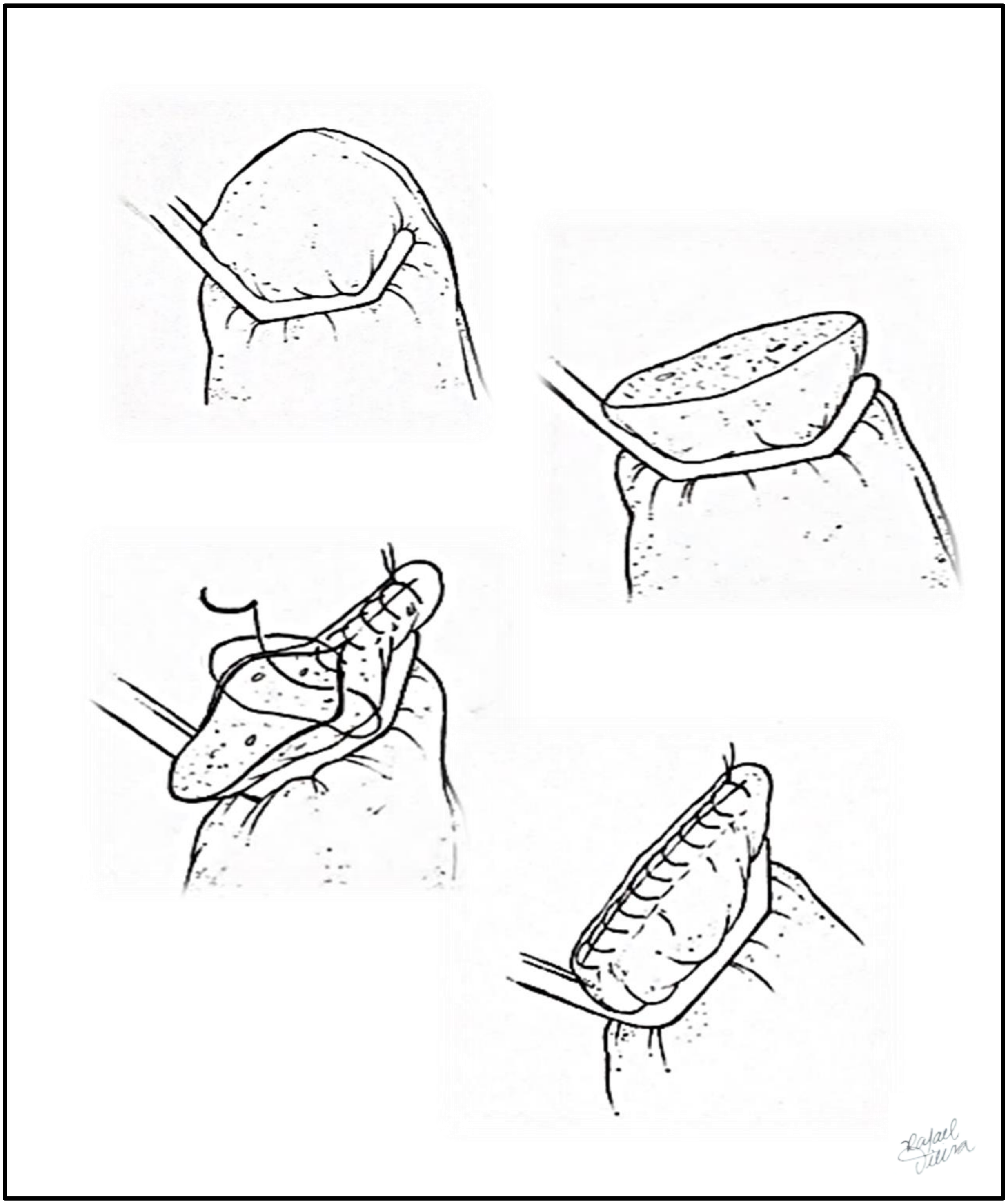

Supplementary Figure S6 - Illustrative drawings demonstrating the steps for making the Ford interlocking suture pattern in the lung lobe of a dog.

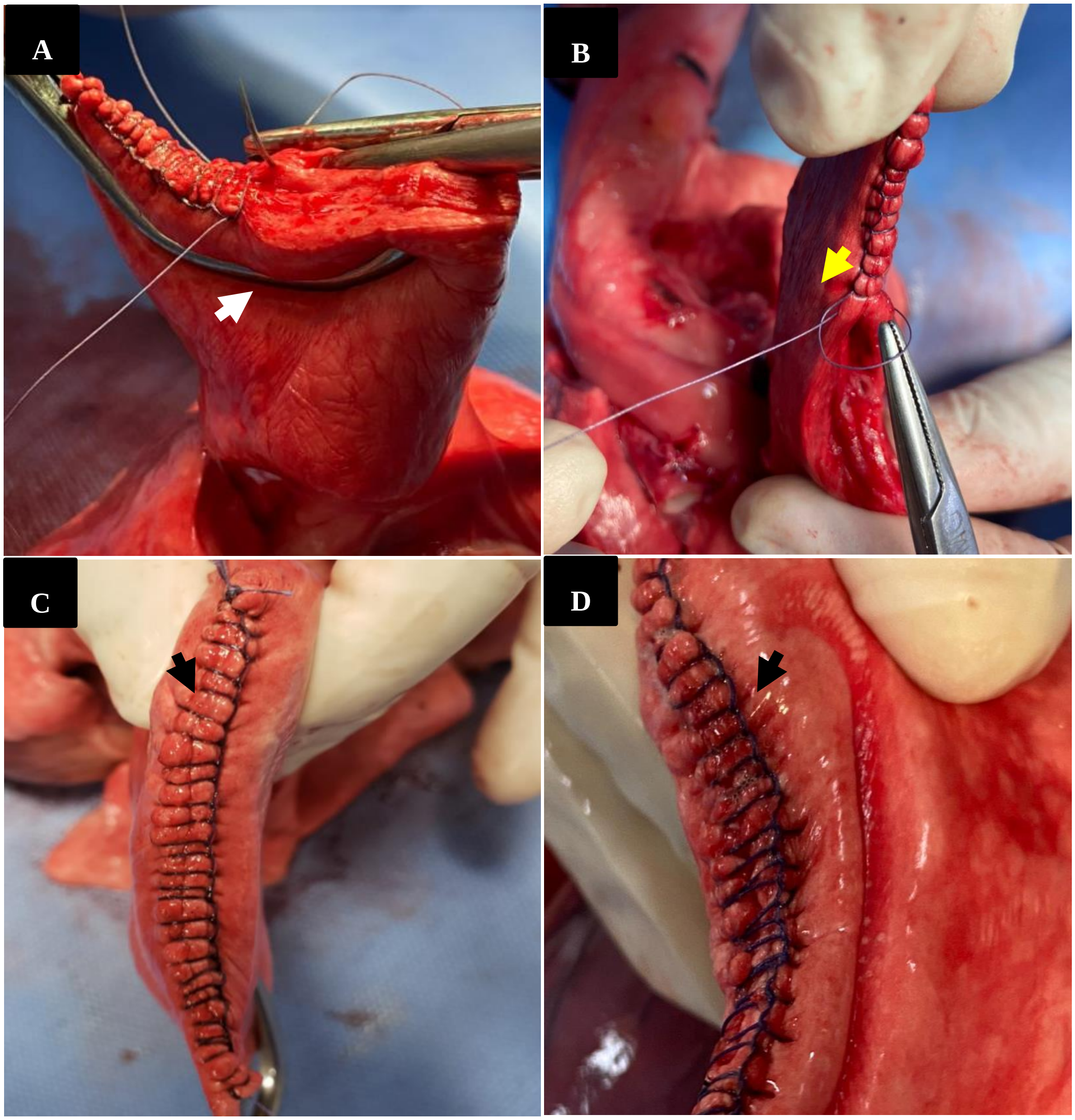

Supplementary Figure S7 - Photographic image of pulmonary suture using Ford interlocking suture pattern in an anatomical piece of dog lung; A and B. Note the clamping with Satinsky forceps (white arrow) in the middle third of the sectioned right caudal lung lobe and making the Reverdin suture loop with Polyglactin 910 thread (yellow arrow). C and D. Final appearance of the parenchyma coaptation and consequently of the visceral pleura after making Ford interlocking suture (black arrows).

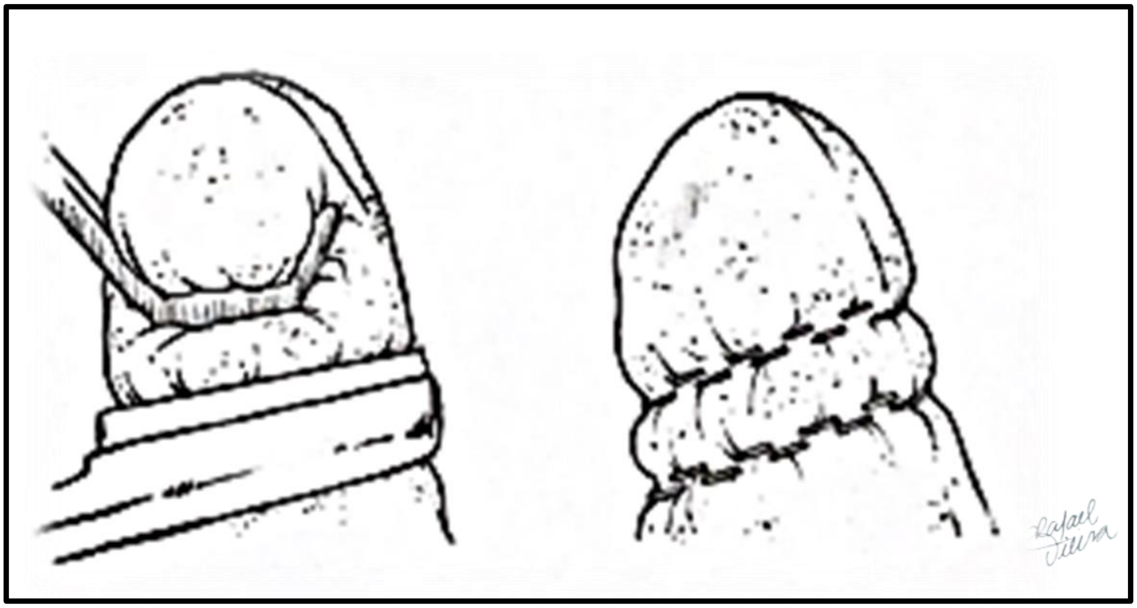

Supplementary Figure S8 - Illustrative drawings demonstrating the steps for making the mechanical suture using the TAsurgical stapler in the lung lobe of a dog.

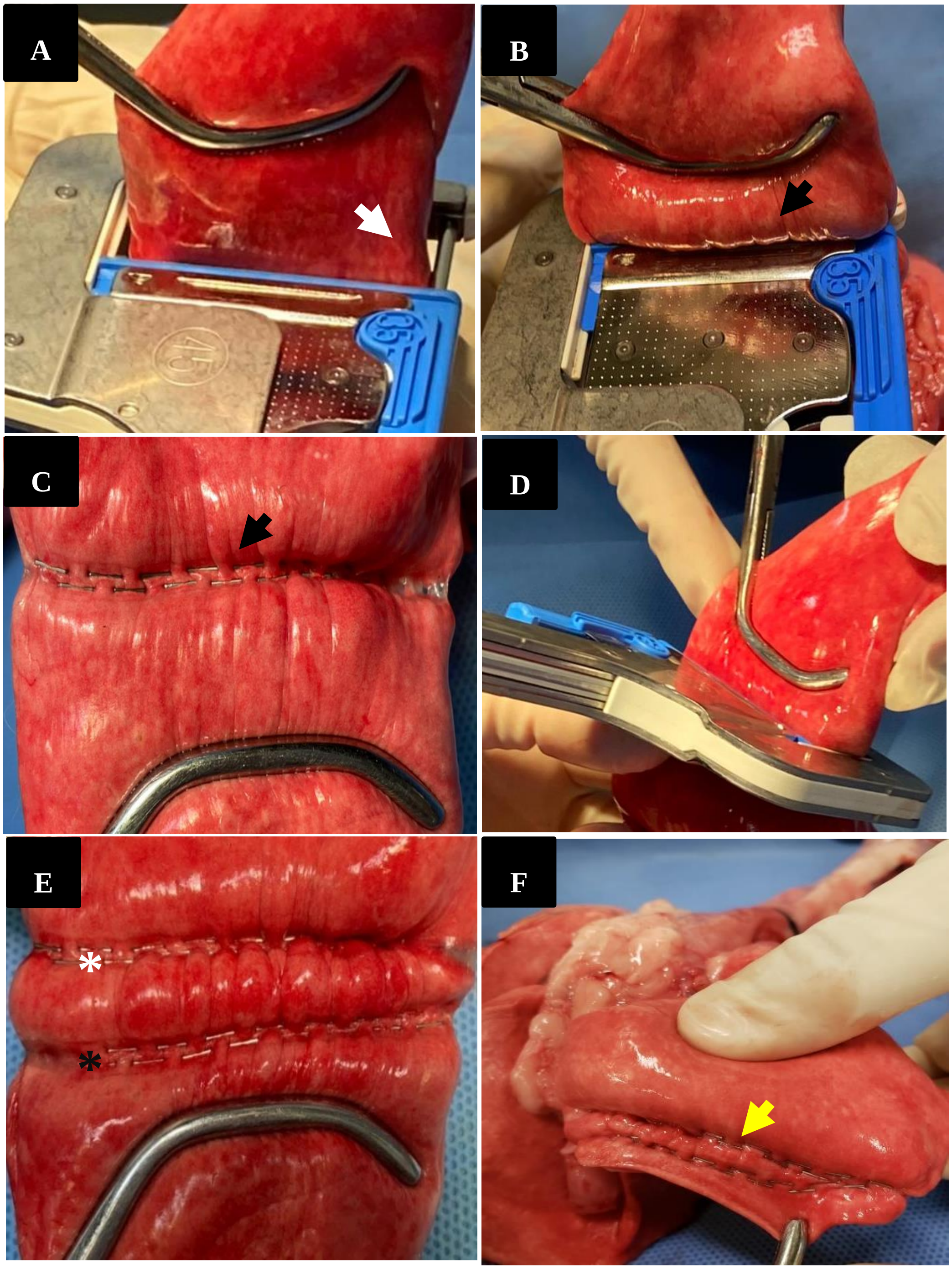

Supplementary Figure S9 - Photographic image of pulmonary synthesis using a TA linear surgical stapler in an anatomical piece of a dog lung. A. Note the clamping in the middle third of the right caudal lung lobe with a Satinsky forceps and introduction of the first cartridge from the lateral region of the lobe and 15 mm proximal to the forceps, followed by the engagement of the retention pin in the anvil (white arrow); B. Compression of the parenchyma followed before the firing of the staples (black arrow); C. The first double row of staples is observed in the parenchyma (blue arrow); D. Device reloaded with second cartridge positioned medially and 5mm proximal to the forceps; E. A second double row of staples

(black asterisk) is observed in the parenchyma after the first (white asterisk); F. Final appearance of the remaining lung lobe after resection between the stapler and the second row of staples (yellow arrow).

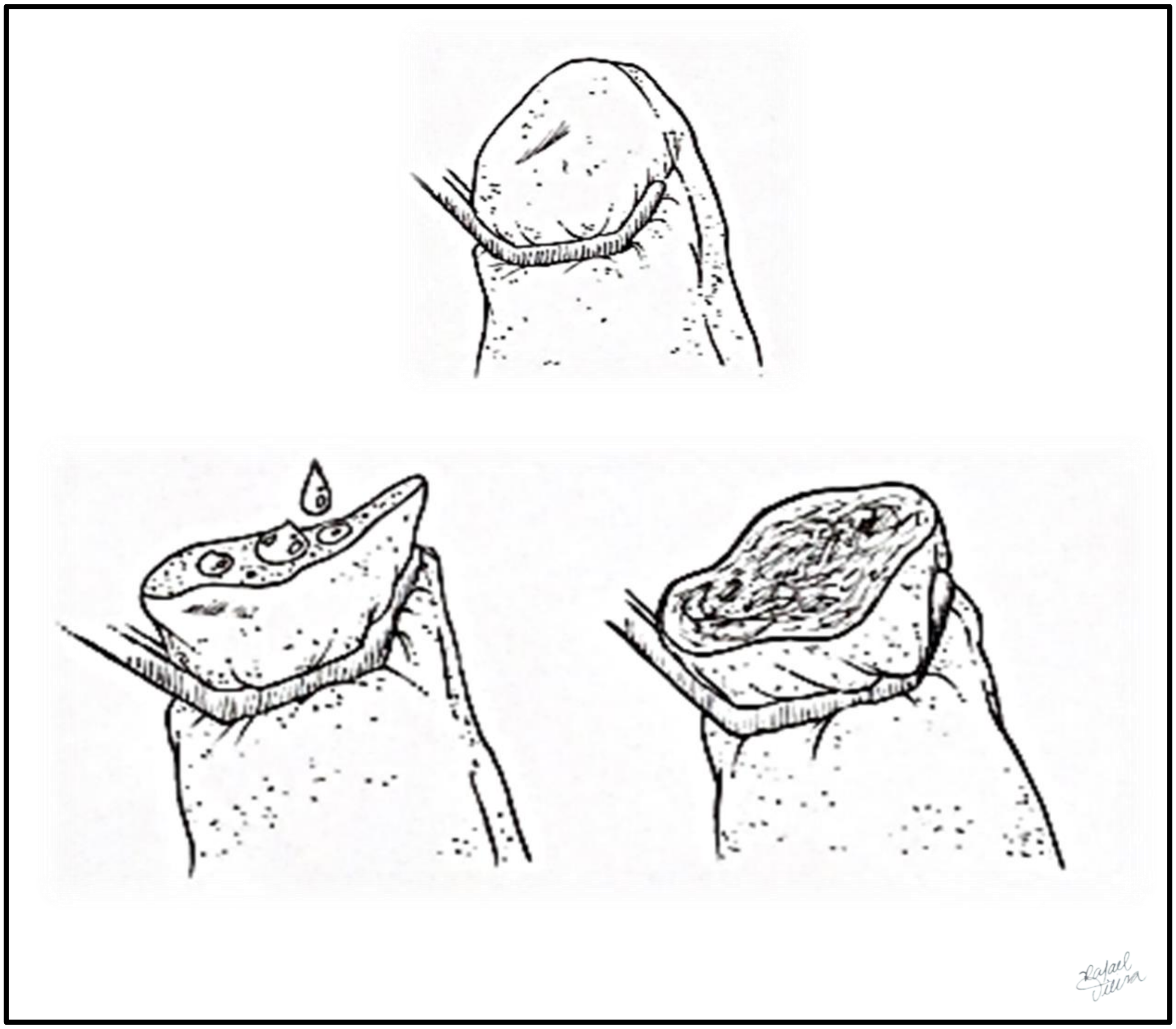

Supplementary Figure S10 - Illustrative drawings demonstrating the steps for making the synthesis of the dog lung lobe with tissue adhesive.

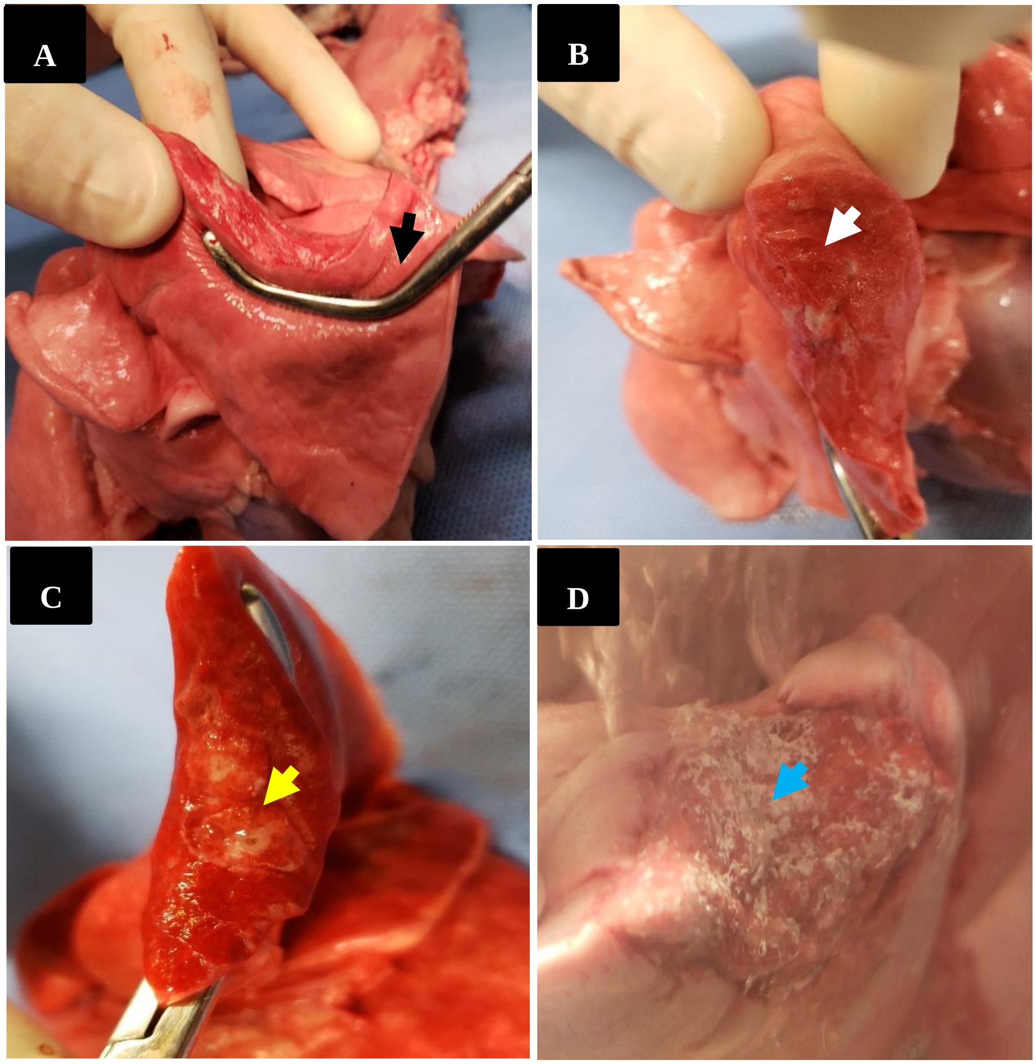

Supplementary Figure S11 - Photographic image of lung sealing using synthetic Glubran-2 adhesive in an anatomical piece of a dog lung; A. Note the clamping in the middle third of the right caudal lung lobe with a Satinsky forceps (black arrow); B. Visualization of the resected parenchyma without the adhesive (white arrow); C. Dry parenchyma is observed with the presence of sealant in bronchioles (yellow arrow) and microvessels; D. Adhesive forming a diffuse whitish film over the resected parenchyma during the aerostasis test (blue arrow).

ANNEX – Document Protocol Commission on Ethics in Animal Use (CEUA)

UFMG

UNIVERSIDADE FEDERAL DE MINAS GERAIS

CEUA
COMISSÃO DE ÉTICA NO USO DE ANIMAIS

Prezado(a):

Esta é uma mensagem automática do sistema Solicite CEUA que indica mudança na situação de uma solicitação.

Protocolo CEUA: 236/2020
Título do projeto: Estudo comparativo da aerostasia promovida por diferentes métodos de síntese após lobectomia pulmonar parcial em cães – Modelo cadavérico (Projeto de Pesquisa)
Finalidade: Pesquisa
Pesquisador responsável: Rodrigo dos Santos Horta
Unidade: Escola de Veterinaria
Departamento: Departamento de Clinica e Cirurgia Veterinaria

Situação atual: Decisão Final - Aprovado

Aprovado na reunião on-line do dia 07/12/2020. Validade: 07/12/2020 à 06/12/2025
Belo Horizonte, 07/12/2020.

Atenciosamente,

Sistema Solicite CEUA UFMG
https://aplicativos.ufmg.br/solicite_ceua/

Universidade Federal de Minas Gerais
Avenida Antônio Carlos, 6627 – Campus Pampulha
Unidade Administrativa II – 2º Andar, Sala 2005
31270-901 – Belo Horizonte, MG – Brasil
Telefone: (31) 3409-4516
www.ufmg.br/bioetica/ceua - cetea@prpq.ufmg.br

UFMG

UNIVERSIDADE FEDERAL DE MINAS GERAIS

CEUA
COMISSÃO DE ÉTICA NO USO DE ANIMAIS

CERTIFICADO
Certificamos que o projeto intitulado "Estudo comparativo da aerostasia promovida por diferentes métodos de síntese após lobectomia pulmonar parcial em cães – Modelo cadavérico (Projeto de Pesquisa)", protocolo do CEUA: 236/2020 sob a responsabilidade de Rodrigo dos Santos Horta que envolve a produção, manutenção e/ou utilização de animais pertencentes ao filo Chordata, subfilo Vertebrata (exceto o homem) para fins de pesquisa científica (ou ensino) - encontra-se de acordo com os preceitos da Lei nº 11.794, de 8 de outubro de 2008, do Decreto nº 6.899 de 15 de julho de 2009, e com as normas editadas pelo Conselho Nacional de Controle da Experimentação Animal (CONCEA), e foi aprovado pela COMISSÃO DE ÉTICA NO USO DE ANIMAIS (CEUA) DA UNIVERSIDADE FEDERAL DE MINAS GERAIS, em reunião de 07/12/2020.

Vigência da Autorização	07/12/2020 a 06/12/2025
Finalidade	Pesquisa
***Espécie/linhagem**	Banco de amostras / SEM RAÇA DEFINIDA
Nº de animais	5
Peso/Idade	15kg / 4(anos)
Sexo	indiferente
Origem	HV UFMG/ CCZ BH
***Espécie/linhagem**	Banco de amostras / SEM RAÇA DEFINIDA
Nº de animais	5
Peso/Idade	15kg / 4(anos)
Sexo	indiferente
Origem	HV UFMG/ CCZ BH
***Espécie/linhagem**	Banco de amostras / SEM RAÇA DEFINIDA
Nº de animais	5
Peso/Idade	15kg / 4(anos)
Sexo	indiferente
Origem	HV UFMG/ CCZ BH
***Espécie/linhagem**	Banco de amostras / SEM RAÇA DEFINIDA
Nº de animais	5
Peso/Idade	15kg / 4(anos)
Sexo	indiferente
Origem	HV UFMG/ CCZ BH
***Espécie/linhagem**	Banco de amostras / SEM RAÇA DEFINIDA
Nº de animais	5
Peso/Idade	15kg / 4(anos)
Sexo	indiferente
Origem	HV UFMG/ CCZ BH
***Espécie/linhagem**	Banco de amostras / SEM RAÇA DEFINIDA
Nº de animais	5

Peso/Idade	15kg / 4(anos)
Sexo	indiferente
Origem	HV UFMG/ CCZ BH
***Espécie/linhagem**	Banco de amostras / SEM RAÇA DEFINIDA
Nº de animais	5
Peso/Idade	15kg / 4(anos)
Sexo	indiferente
Origem	HV UFMG/ CCZ BH

Considerações posteriores:

07/12/2020	Aprovado na reunião on-line do dia 07/12/2020.<br>Validade: 07/12/2020 à 06/12/2025

Belo Horizonte, 07/12/2020.

Atenciosamente,

Sistema Solicite CEUA UFMG
https://aplicativos.ufmg.br/solicite_ceua/

Universidade Federal de Minas Gerais
Avenida Antônio Carlos, 6627 – Campus Pampulha
Unidade Administrativa II – 2º Andar, Sala 2005
31270-901 – Belo Horizonte, MG – Brasil
Telefone: (31) 3409-4516
www.ufmg.br/bioetica/ceua - cetea@prpq.ufmg.br
